# Characteristics of Reservoir Boundary Ranging with While-Drilling Impulse Sound Source

**DOI:** 10.3390/s26031035

**Published:** 2026-02-05

**Authors:** Haiyan Shang, Sen Gao

**Affiliations:** 1 Downhole Measurement & Control Laboratory, National Engineering Research Center of Oil & Gas Drilling and Completion Technology, Xi’an Shiyou University, Xi’an 710065, China; 24211030414@stumail.xsyu.edu.cn; 2 School of Electronic Engineering, Xi’an Shiyou University, Xi’an 710065, China

**Keywords:** impulse sound source, rotating parabolic reflector, reservoir boundary ranging, energy bunching, ranging precision

## Abstract

Reservoir boundary distance measurement is a key technology in geosteering drilling. In this field, it is difficult to balance detection precision and depth. This paper proposes a method to measure reservoir boundary distance using a drill-attached impulse sound source equipped with a reflector. The COMSOL Multiphysics (COMSOL) is used to construct a while-drilling reservoir model with a reflector and verify the model’s effectiveness through the real-axis integration method. Under this model, the dimensions of the reflector are analyzed, the relative ranging error under different distances is calculated, and source distance combinations and reservoir interface dip angles are considered. Moreover, the effectiveness of this method is verified through the results of ranging for two sets of actual geological parameters. These results show that the rotating parabolic reflector (depth 45 mm, opening radius 12.2 mm) has a good energy bunching effect. When the dominant excitation frequency of the sound source is 8 kHz, and the source distance combination is 2 m and 4 m, the minimum relative ranging error for the reservoir boundary at 7 m is 2.1%. The relative error becomes smaller when the reservoir boundary dip angle and source distance are smaller. When the source distance is 2 m or 7 m, and the dip angle is between [−20, 20] degrees, the relative error is below 15%. Simulations with actual formation parameters indicate that the proposed method attains good ranging precision.

## 1. Introduction

With the progress of modern oil and gas exploration technology, more and more oil and gas reservoirs have been proven. However, we also face the problem of easy exploration but difficult production [1]. Most of China’s proven oil and gas resources are unconventional oil and gas reservoirs that are difficult to exploit, such as many large oil and gas fields in regions including Ordos, Bohai Bay, Songliao, Junggar, and Sichuan [2,3]. Internationally, oil and gas exploitation also focuses on unconventional oil and gas reservoirs [4,5,6,7]. Although China has proven huge reserves of unconventional oil and gas reservoirs in recent years, there is still a gap between its drilling and production technologies and equipment and the world’s leading level [2]. This leads to the production cost of crude oil being higher than the price of imported crude oil [8]. In the development of unconventional oil and gas reservoirs, using rotary steerable geosteering technology while drilling can hit “sweet spots” with a smooth wellbore and extend long distances within reservoir blocks, thereby significantly improving the oil and gas recovery rate [9]. Therefore, the independent industrial chain of rotary steerable geological steering technologies and equipment can remarkably enhance oil and gas exploitation efficiency and oil and gas production, which is of great significance for the development of the petroleum industry.

Geosteering while-drilling technology is essentially a closed-loop system for real-time data collection, data interpretation, decision-making, and control during the drilling process. It includes measurement-while-drilling technology and constructs and updates geosteering models. The main methods of measurement-while-drilling are logging-while-drilling, seismic-while-drilling, and real-time formation-pressure monitoring during drilling. The main measurement parameters of logging-while-drilling include resistivity to identify oil and gas layers [10], gamma rays to identify lithology [11], neutron porosity and density to evaluate formations [12], and acoustic velocity for rock mechanics analysis and seismic data calibration [13]. Seismic-while-drilling enables long-distance geological prediction [14]. Real-time formation-pressure monitoring during drilling mainly monitors the parameters for safe drilling and optimizes steering decisions. Constructing and updating geosteering models involves analyzing, modeling, and making decisions based on the measured data. The conventional method for achieving this is based on the initial geological model. By supplementing actual drilling data such as gamma and resistivity curves, the geological model is adjusted and updated, for example, by modifying formation interfaces and reservoir thickness. Then, based on the updated model, the geological conditions ahead of and around the drill bit are predicted, and suggestions for adjusting the wellbore trajectory are provided. The real-time intersection analysis of multi-information, as well as integrated cross-analysis and verification of geological, engineering, and geophysical information, can enhance the user’s capability to make decisions [15].

A combination of near-bit measurement with boundary detection is one of the new geosteering technologies. Sensors are installed as close to the drill bit as possible, and technologies such as directional acoustic waves or electromagnetic waves are used to detect reservoir boundaries within several meters or more than ten meters during drilling. This makes it possible to determine the direction and distance of the boundaries and guide the wellbore trajectory to navigate within the reservoir [16]. For example, acoustic far-detection logging uses a sound source to excite acoustic waves, which reflect when they encounter discontinuous acoustic impedance such as fractures, faults, or formation interfaces outside the wellbore. The receiving array receives the reflected acoustic wave energy, extracts the reflected wave signals, and then processes and analyzes the signals to understand the geological structures around the wellbore [17].

According to the difference in the type of excited sound source, there are mainly three types of deep detection technologies: monopole, dipole, and multipole [18]. A monopole sound source is typically implemented as a pressure pulse source in a wellbore, which radiates spherical waves outward in all directions and thus lacks azimuth recognition capability [19]. A dipole sound source can be equivalent to two points of sound distributed 180° apart with opposite phases, achieving a maximum detection distance of 70 m at low frequencies. However, it suffers from 180° azimuth ambiguity, and its detection resolution at low frequencies is relatively low [20]. Among multipole sound sources, the quadrupole sound source, for instance, can be equivalent to four points of sound distributed 90° apart with alternating phases. It exhibits strong azimuthal directivity, but its transducer structure is complex, and its energy radiation efficiency is relatively low [21].

This research uses an impulse sound source as the while-drilling detection source. The principle of the impulse sound source is to store energy with a large capacity energy storage capacitor and then to release high voltage impulses instantaneously in the water medium through the gap between the discharge electrodes. Previously, in a verification of a physical impulse sound source and energy bunching device conducted by X H et al. [22], these were found to exhibit the characteristics of high power, a wide frequency band, and repeatability and controllability. By equipping a rotating paraboloidal reflector, the impulse sound source can gain directivity and form an energy bunching effect, thereby achieving higher excitation energy. This gives it certain advantages in its detection depth and ranging precision, helping to accurately detect the reservoir location and, further, to guide the adjustment of the wellbore trajectory to obtain a higher drilling encounter rate. Based on the while-drilling impulse sound source, the COMSOL Multiphysics (COMSOL 6.1) numerical simulation software is adopted to investigate the reflector dimensions suitable for reservoir ranging with 7-inch drill collars, as well as their ranging effects under different reservoir boundary distances, source distance combinations, reservoir interface dip angles, and actual formation parameters. The effectiveness of this method is verified through the real-axis integration method. This paper verifies the capability of the impulse sound source for reservoir boundary ranging at the numerical simulation level, thus laying a certain research foundation for subsequent physical experiments on impulse sound source ranging and the future development of logging while drilling (LWD) equipment based on impulse sound sources.

## 2. Principle and Characteristic Analysis of Rotating Paraboloidal Reflector

### 2.1. Principle and Model of Rotating Paraboloidal Reflector

**Figure 1:**
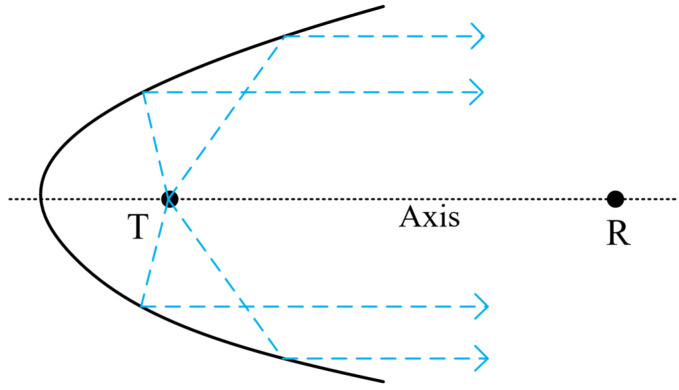
Schematic diagram of the rotating paraboloidal reflector model.

Reflective acoustic energy bunching mainly uses the principle of curved baffle reflection to achieve the purpose of bunching spherical waves generated by impulse sound sources [23]. According to the geometric model of the reflector, it is usually divided into rotating ellipsoidal reflectors and rotating paraboloidal reflectors. After reflecting the spherical wave field, the ellipsoidal reflector converges it to a single point, that is, it focuses the wave field generated by the sound source, and achieves the best effect when detecting reservoir boundaries at specific distances [24]. After reflecting the spherical wave field, the rotating paraboloidal reflector forms parallel beams, which not only achieves an energy bunching effect but also ensures that the generated reflected waves are not excessively restricted. It can be applied to the detection of reservoir boundaries at any distance [25]. In this research, the reflector structure is used for reservoir boundary ranging near the wellbore. Since the thickness and boundaries of the reservoir are unknown, a rotating paraboloid is adopted as the reflector structure of the impulse sound source. Its schematic diagram is shown in Figure 1. The impulse sound source is placed at the focal point *T* of the paraboloid. Part of the acoustic pulse energy on the right side of the impulse sound source propagates outward in the form of spherical diffusion, and the remaining pulse energy is reflected by the paraboloidal reflector and then propagates parallel to the direction of the reflected waves of the reflector. The receiver is set at position *R*.

**Figure 2:**
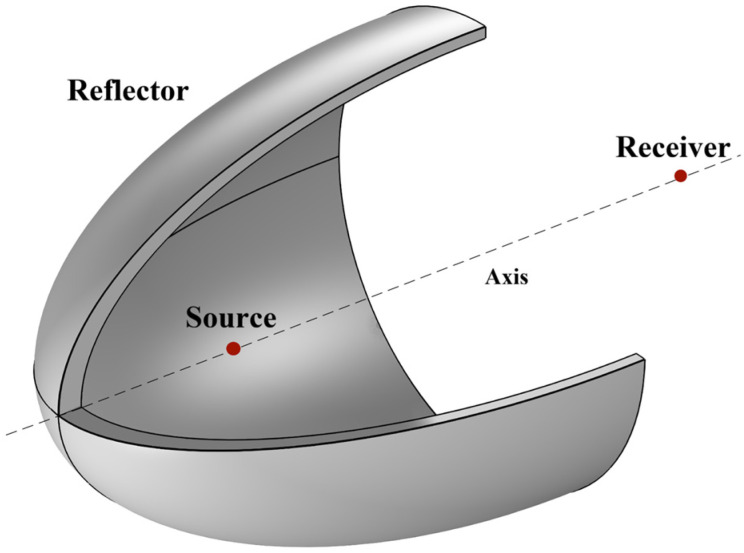
Reflector simulation model.

The reflector model is constructed using the pressure acoustic field in COMSOL. A three-dimensional model is adopted for model construction, and the underwater model of the reflector is shown in Figure 2. The model consists of two parts: the reflector and water. It is a cube with a side length of 2 m. To better observe the energy bunching effect of the reflector and eliminate the influence of boundaries on the simulation, the boundary condition of the model is set as a PML (Perfectly Matched Layer), and the reflector uses an internal hard sound field boundary.

The while-drilling impulse detection source adopts the plasma impulse sound source developed by the Downhole Measurement and Control Laboratory of Xi’an Shiyou University.

**Figure 3:**
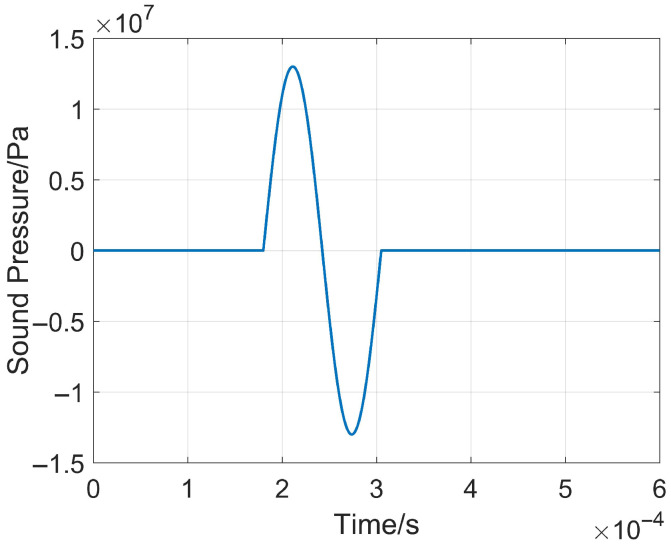
Impulse sound source fitting function curve.

This sound source has a wide frequency band of 0~100 kHz. Based on the reference sound pressure in water ($P_{ref}=1\times 10^{-6}\;Pa$), it can reach a maximum sound pressure level of 265 dB, as well as controllable and repeatable excitation [22,26]. Experimental data are fitted by using MATLAB R2024a, and the time-domain expression of the plasma impulse sound source function is obtained as shown in Equation (1) [27]:

(1) $$s(t)=A\sin\left[2\pi f_{0}\left(t-t_{0}\right)\right]rect\left(f_{0}\left(t-t_{0}\right)-0.5\right)$$

where the amplitude $A=1.3\times 10^{7}$, the electrode pre-breakdown time $t_{0}=1.8\times 10^{-4}\;s$, and the dominant excitation frequency of the sound source is $f_{0}$. To balance detection depth and detection resolution, the dominant frequency $f_{0}$ is taken as 8 kHz and the corresponding function curve is shown in Figure 3.

To verify the reliability of the numerical simulation adopted in this paper, the fluid-filled borehole sound field in an isotropic medium is taken as the verification object, and the calculation results of the numerical simulation and real-axis integration (an analytical calculation method for borehole sound field) are compared and analyzed [28]. See Appendix A for the derivation process of the time-domain analytical solution of the axial sound field of the parabolic reflector. A COMSOL model with a size of 4 m × 11 m is established, and the parameters corresponding to the 7in drill collar are as follows: the radius of the fluid inside the drill collar is 0.0357 m, the radius of the drill collar is 0.0889 m, and the radius of the borehole is 0.10795 m. The minimum source distance to the receivers is 1 m. Without a loss of generality, the received waveforms of 3 receivers with an interval of 1 m are calculated, and the dominant frequency of the impulse sound source is still 8 kHz.

**Figure 4:**
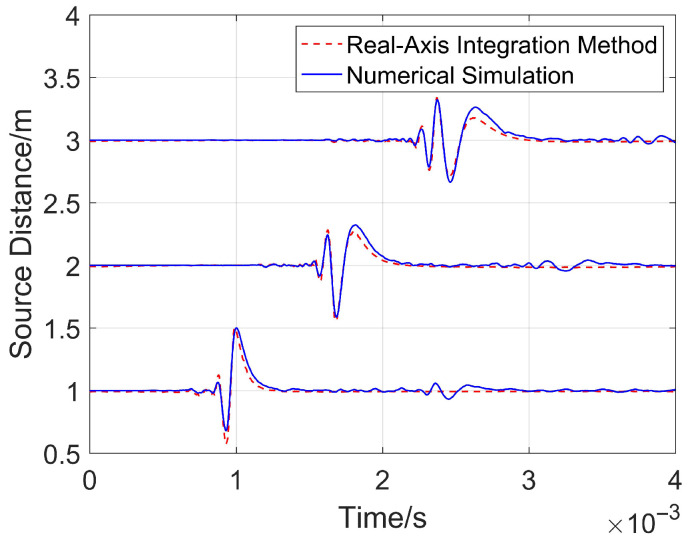
Comparison of real-axis integration and numerical simulation.

Figure 4 shows the calculated comparative waveforms. It can be seen that the two curves have a high degree of overlap under different source distances. The root mean square errors (RMSE) of the waveforms received by the three receivers, calculated from the nearest to the farthest, are 0.0484, 0.0463, and 0.0466, respectively. This indicates that the results obtained by numerical simulation are in good agreement with the analytical solutions derived from the real-axis integration method. This verifies that the numerical simulation method adopted in this paper is reliable, and can be used to calculate complex two-dimensional numerical models, including tight reservoirs, horizontal boreholes, and formation interfaces.

### 2.2. Energy Bunching Effect of Reflector Depth and Caliber Size

Due to the limited installation space on the drill collar, the size of the reflector cannot be too large. On the premise of meeting the size requirements of the drill collar to obtain the best possible energy bunching effect, the selection of the reflector’s depth and caliber is analyzed as follows. In this section, the energy bunching effect of energy bunching devices of different dimensions is investigated by comparing the maximum value of the waveform received by the receivers, i.e., the sound pressure peak. The rotating parabola function shown in Figure 3 is as shown in Equation (2):

(2) $$\left\{\begin{array}{c} x=\frac{1}{2p}y^{2}\\ T\left(\frac{p}{2},0\right) \end{array}\right.$$

where *x* and *y* are the horizontal and vertical coordinates, corresponding to the reflector’s depth *B* and opening radius *r*, respectively.

**Figure 5:**
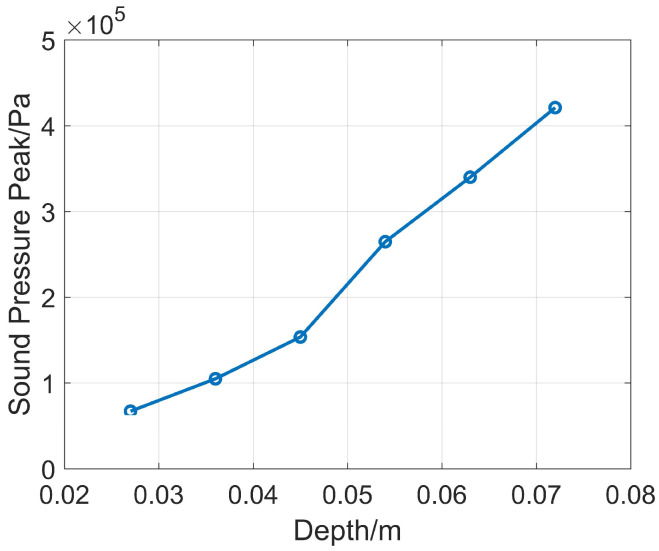
Comparison of sound pressure peak values of reflectors with the same opening radius and different depths.

First, the opening radius is fixed at 30 mm, and only the depth structure of the reflector is changed. The value of $\frac{1}{2p}$ is selected in the range of 30~80 mm, with a step size of 10 mm, corresponding to the reflector depths B of 27 mm, 36 mm, 45 mm, 54 mm, 63 mm, and 72 mm. Numerical simulations are carried out under this structure, and the peak value comparison of the received sound pressure waveforms is shown in Figure 5. It can be seen that when the opening radius *r* is constant, the reflector depth is larger and the energy bunching effect is better. To balance the mechanical strength of the installed drill collar and the energy bunching effect in the directional direction, the reflector depth *B* is selected as 45 mm in subsequent simulations.

**Figure 6:**
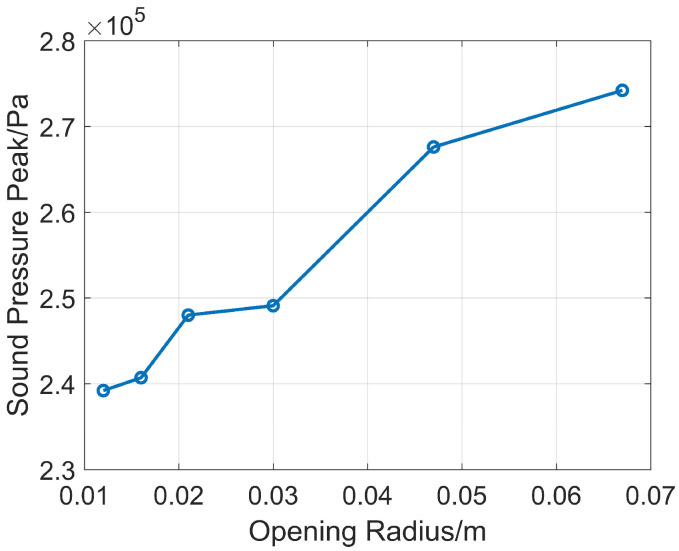
Comparison of sound pressure peak values of reflectors with the same depth and different opening radius.

When the depth is 45 mm, the opening radii *r* are 12.2 mm, 15.8 mm, 21.2 mm, 30 mm, 47.4 mm, and 67.1 mm. Numerical simulations are carried out, and the sound pressure peak values obtained through comparison are shown in Figure 6.

Figure 6 shows that the larger the opening radius, the higher the sound pressure peak value. Although other opening radii are several times larger than 12.2 mm, the increase in sound pressure peak value is not obvious. The sound pressure peak difference between the smallest and largest opening radii is only 1.186 dB. It shows that the opening radius has little influence on the sound pressure peak value. Therefore, considering drilling safety, the opening radius *r* of the reflector is selected as 12.2 mm.

### 2.3. Verification of Energy Bunching Effect of Reflector

**Figure 7:**
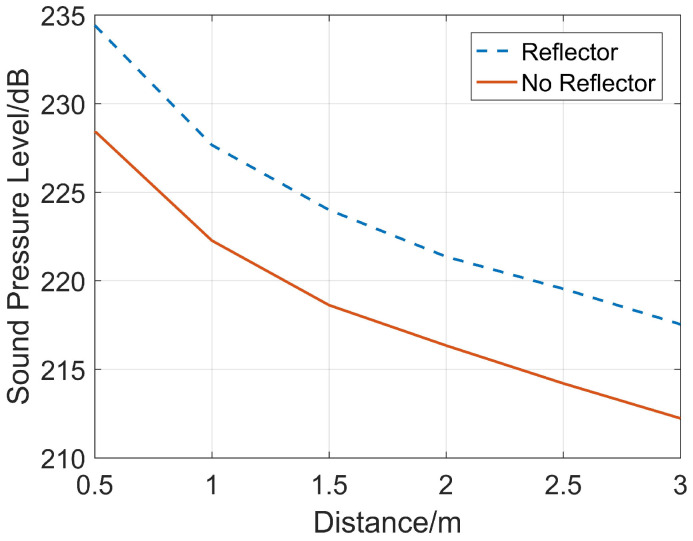
Comparison of the energy bunching effect of the reflector.

After selecting the reflector size, a three-dimensional model is established. On the axis of the reflector, six receiving points with a spacing of 0.5 m are set in sequence 1 m away from the transmitting source. Simulations are carried out for two cases (with and without the reflector). Based on the reference sound pressure in water ($P_{ref}=1\times 10^{-6}\;Pa$) for sound pressure level calculation, a comparison of the sound pressure levels of the sound pressure waveforms corresponding to the full wave trains received in both cases is shown in Figure 7. After energy is bunched by the paraboloidal reflector, the sound pressure levels of the sound pressure peaks on the reflector’s axis are all greater than those without the reflector. This shows that a rotating paraboloidal reflector of this size enables the impulse sound source to obtain greater incident signal energy, thereby improving the SNR (Signal-to-Noise Ratio) of the received reflected wave signals.

Energy bunching of sound waves is inevitably accompanied by the directivity problem of the sound source. Currently, in the research into azimuthal acoustic detection, related research topics on sound source directivity are gradually increasing in relevance. As a monopole sound source, the impulse sound source has the advantages of high energy and a wide frequency bandwidth. However, this type of monopole point source does not have the ability to radiate directionally to a specific azimuth and is non-directional. Therefore, it is also necessary to investigate the sound wave directivity effect after equipping the energy bunching reflector.

**Figure 8:**
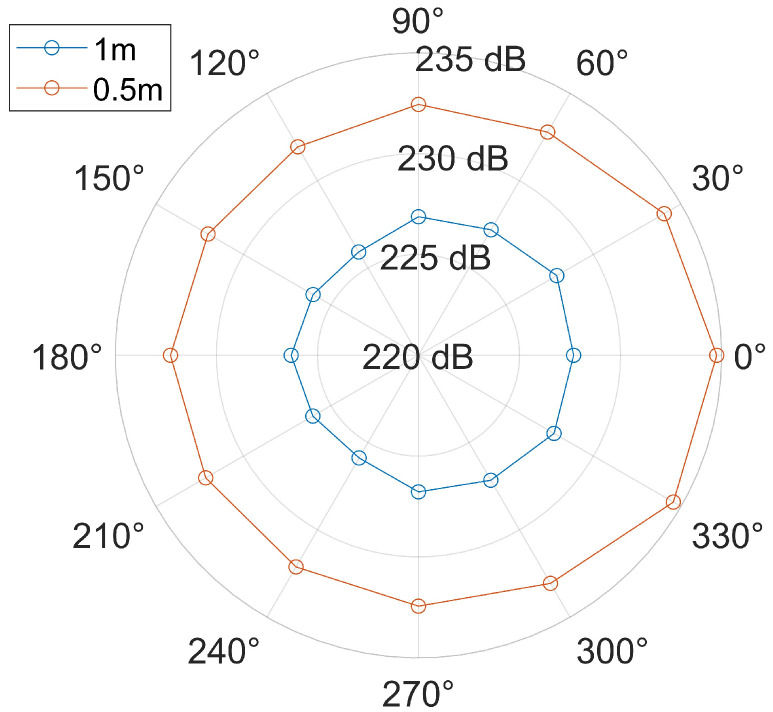
The polar coordinate diagram of the energy bunching directivity effect of the reflector.

As shown in Figure 8, it is assumed that the 0° direction represents the opening direction of the parabolic energy bunching reflector, and the impulse sound source is located at the center. Starting from the 0° direction, 12 receiving points are arranged around the sound source counterclockwise at intervals of 30° at distances of 0.5 m and 1.0 m from the sound source. The calculated sound pressure levels are plotted in Figure 8.

As can be seen from Figure 8, on the one hand, the sound pressure levels of the waveform received by the receiving point in the 0° direction are the largest. That is to say, the impulse sound source can achieve a good directional radiation effect in the opening direction of the paraboloid after being reflected by the parabolic reflector. On the other hand, when the distance between the observation points and the sound source increases, there is a certain attenuation of wave field energy during propagation in the medium, so the sound pressure levels show a decreasing trend. However, the directional radiation effect remains good within a certain distance range.

## 3. Numerical Simulation and Analysis of Reservoir Boundary Ranging Effect near Wellbore

Rotary-steerable drilling technology collects geological structure information around the wellbore during drilling. It then guides the drill collar to drill into the reservoir so as to get a higher oil and gas recovery rate. So, it is necessary to construct and simulate a reservoir boundary model adjacent to the wellbore.

Since the near-wellbore reservoir boundary model involves not only the pressure acoustic field but also the solid mechanics field, we choose COMSOL software, which is more suitable for multiphysics coupling analysis, to establish the while-drilling near-wellbore reservoir boundary model [13,29]. In this section, impulse acoustic waves are used as the detection source to analyze the reflection wave characteristics when the shock wave encounters the interface.

### 3.1. Establishment of the Near-Wellbore Reservoir Boundary Model

The actual logging-while-drilling (LWD) conditions are extremely complex; thus, appropriate simplifications of real engineering scenarios are required during the numerical modeling process. Due to the complex rock compositions of actual reservoirs, formations typically exhibit anisotropic characteristics. In simulations, they are simplified as ideal isotropic linear elastic materials, and numerical solutions are realized by combining the finite element discretization method.

Regarding the selection of model dimensions, 3D models are more realistic and effective, but they consume substantial computer memory, involve heavy computational loads, and require long calculation times. Previously, in Section 2.1, we verified the validity of simulation results under the 2D model by calculating the root–mean–square error (RMSE) between the results of the real-axis integration method and COMSOL simulations. Therefore, to ensure the validity of results while enhancing simulation efficiency, this paper adopts the 2D model for acoustic field simulation.

It should be noted that the simplified 2D model cannot fully represent the spatial structure of the drill collar and formation models, nor can it be used to investigate the spatial orientation characteristics of reservoir boundaries. Moreover, the originally integrated cylindrical drill collar is divided into two separate parts, leading to distortion in the received full wave trains. However, this does not affect the exploration of the physical laws governing reservoir boundary ranging in this study, and the ranging research conducted under the 2D model remains feasible and valid.

**Figure 9:**
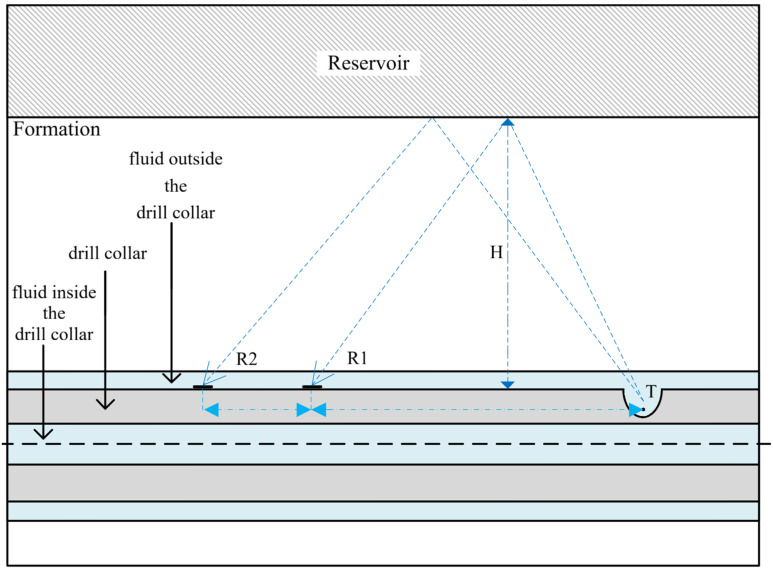
While-drilling near-wellbore reservoir boundary model.

The schematic diagram of the geometric model of the while-drilling near-wellbore reservoir boundary is shown in Figure 9. From the inside out, the geometric model consists of the fluid inside the drill collar, the drill collar, the fluid outside the drill collar, the formation, and the oil-bearing reservoir above the model in sequence. Both the fluids inside and outside the drill collar are filled with water, the drill collar is made of non-magnetic drill collar steel, and the distance between the reservoir boundary and the drill collar is *H*. The impulse sound source is placed at the focal point *T* of the reflector, and the energy bunching direction points to the reservoir, as shown in Figure 9.

To simplify the model, the receivers mounted on the drill collar were configured as domain point probes in COMSOL, and this study investigated the total sound pressure received by them. The model parameters and dimensions used in the calculation are shown in Table 1 [27]. The height of the model is set to 11 m, and its width is related to the distance *H* from the reservoir boundary.

The Pressure Acoustics (Transient) and Solid Mechanics physics interfaces are used, and Acoustic–Structure Interaction (Transient) for multiphysics coupling is selected. In the actual logging process, formations are generally regarded as an infinite linear elastic medium. To simulate this characteristic, the boundary of the Pressure Acoustics field in the model is set as plane wave radiation, and the boundary of the Solid Mechanics field is configured as a low reflection boundary.

The fluid inside and outside the drill collar was set to the Pressure Acoustics physics interface, while the remaining parts were configured as Solid Mechanics. The Acoustic–Structure Boundary was adopted for the interface between these two physics fields. Since the acoustic impedance of oil-bearing reservoirs differs from that of the formation, reflected waves are generated when acoustic waves pass through their interface, and thus, no additional settings are required for this interface.

The solution accuracy of the finite element discretization method is related to the meshing size. The finer the mesh, the closer the obtained solution is to the analytical solution; however, this increases the degrees of freedom of the model and thus prolongs the calculation time. For acoustic wave propagation problems, approximately 10 or 12 nodes are required per wavelength to accurately resolve the wave, i.e., at least 5 s-order mesh elements should be used within a single wavelength. Receivers are set starting from 1 m away from the sound source. Free triangular elements were adopted for meshing, with a maximum element size of λ/5 and a minimum element size of λ/8, where λ denotes the wavelength corresponding to the excitation dominant frequency of the sound source [27]. The time step was set as $\Delta t=1/\left(60f_{0}\right)$, where $f_{0}$ is the selected excitation dominant frequency of the sound source, set at 8 kHz. Considering the robustness and the operating memory capacity of the computer, the MUMPS direct solver was chosen for the numerical solution. A total of seven receivers are installed with an interval of 1 m, placed on the drill collar wall, and two receivers are illustrated in Figure 9.

### 3.2. Ranging Effect of Near-Wellbore Reservoir Boundary at Different Distances

Due to the limited build rate of rotary steering tools in actual drilling, for example, when using point drill bit rotary steering, the maximum build rate of Schlumberger’s rotary steering system is approximately 8.0°/30 m [30]. Therefore, the equipment needs to have the ability to detect reservoir boundaries at an appropriate distance so as to improve the hit rate of oil and gas. In this way, it can guide the adjustment of the wellbore trajectory direction in advance.

When the P-wave and S-wave velocities of the formation are unknown, the straight-line distance from the reservoir boundary to the drill collar is calculated by Equation (3):

(3) $$H=\sqrt{\frac{t_{1}^{2}x_{2}^{2}-t_{2}^{2}x_{1}^{2}}{4t_{2}^{2}-4t_{1}^{2}}}$$

where *H* is the distance from the reservoir boundary to the drill collar, $t_{1}$ is the reflection wave arrival time at receiving point $R_{1}$, $t_{2}$ is the reflection wave arrival time at receiving point $R_{2}$, $x_{1}$ is the source distance of receiving point $R_{1}$, and $x_{2}$ is the source distance of receiving point $R_{2}$.

**Figure 10:**
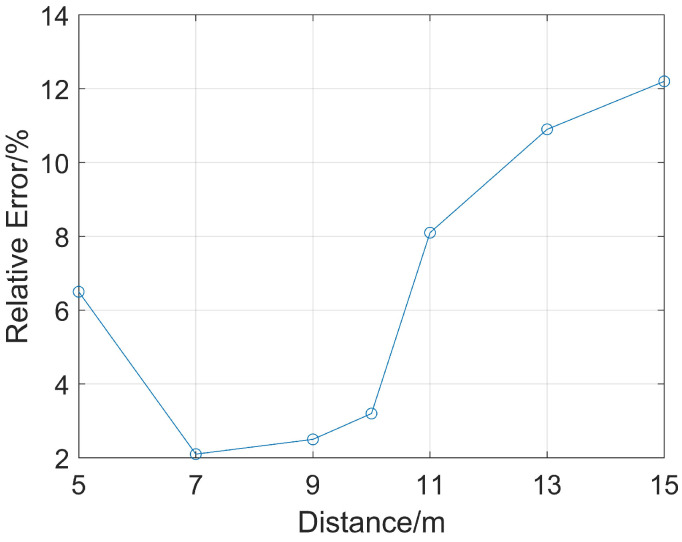
Relative error of ranging at different distances when the excitation dominant frequency of the sound source is 8 kHz.

Receiving points with source distances of 2 m and 4 m are selected to analyze the ranging accuracy when the distance from them to the reservoir boundary ranges from 5 m to 15 m with an interval of 2 m. Due to the existence of the drill collar, drill collar mode waves are generated. Such waves propagate along the inside of the drill collar from the sound source, with relatively large sound pressure and a faster wave velocity than the reflected waves in the formation. In the full wave train, the reflected waves are completely submerged by the drill collar mode waves and cannot be directly observed. Therefore, it is necessary to first extract the reflected waves from the full wave train signals. To completely eliminate all interference signals and obtain intact reflected wave signals, this study first removed the oil-bearing reservoir for simulation, yielding full wave trains without reflected wave signals. At this point, the full wave train signals only contained interference signals such as drill collar mode waves. Subsequently, simulations were conducted based on the complete model, and the obtained full wave train signals were subtracted from the full wave trains without reflected wave signals, thereby extracting intact and interference-free reflected wave signals. For the determination of wave arrival time, this study adopted the method of extracting the peak value of the acoustic wave envelope, which was used for subsequent research. Then, when the P-wave and S-wave velocities of the oil-bearing reservoir are unknown, Equation (3) is used to calculate the straight-line distance from the sound source to the reservoir boundary. The error between this calculated distance and the known boundary distance is obtained, and the relative error of the ranging accuracy is calculated as shown in Figure 10.

As shown in Figure 10, the relative error first decreases and then increases. When the reservoir boundary distance is 7 m, the error is the smallest, only 2.1%. So, the following research uses 7 m as the reservoir boundary distance to carry on.

In sonic logging, as the frequency decreases, the wavelength increases, and its penetration ability becomes stronger, but the resolution also decreases. Therefore, if we want to detect the reservoir boundary at a greater distance with high accuracy, there is a contradiction in the requirements for the dominant frequency of the acoustic source. Higher accuracy requires a higher frequency, while long-range detection requires a lower frequency. The wide-frequency characteristic of the impulse sound source can alleviate this contradiction. Based on the previous research results [31], we integrate the requirements for the detection distance and accuracy of the reservoir boundary around the well, and conduct distance measurement with the dominant frequency of the impulse sound source at 8 kHz.

### 3.3. Ranging Effect of Different Source Distance Combinations

**Figure 11:**
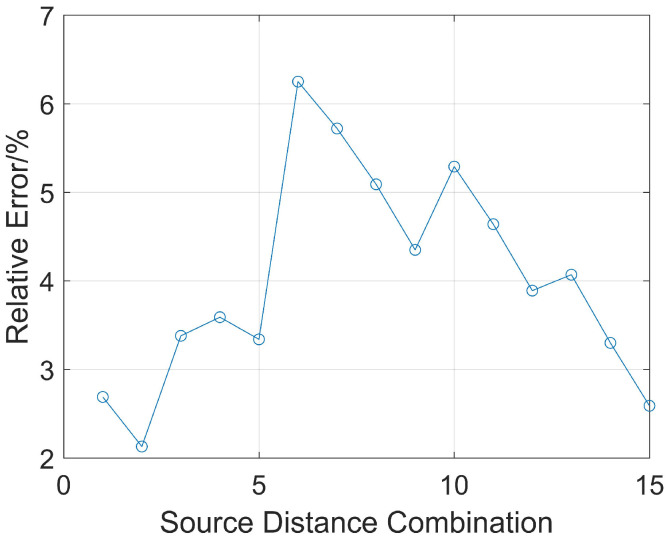
Relative error of ranging for different source distance combinations.

As shown in Equation (3), calculating the boundary distance requires the reflection wave arrival times and source distances of two receiving points. Next, the relative error of ranging accuracy for different source distance combinations of receiving points is analyzed. The received signals from six receivers (with source distances ranging from 2 m to 7 m) are selected and are used in their pairwise combinations to calculate the relative error of ranging accuracy when the reservoir boundary distance is 7 m. There are Group $C_{6}^{2}$ source distance combinations. For example, two receivers with source distances of 2 m and 3 m form a group to calculate the ranging distance, noted as (2, 3). The 15 combinations are sorted in sequence as follows: (2, 3), (2, 4), (2, 5), (2, 6), (2, 7), (3, 4), (3, 5), (3, 6), (3, 7), (4, 5), (4, 6), (4, 7), (5, 6), (5, 7), and (6, 7). The relative error of ranging is shown in Figure 11.

When the second source distance combination of 2 m and 4 m is used, the relative error of ranging accuracy is the smallest at 2.1%. When the sixth combination, 3 m and 4 m, is used, the relative error is the largest at 6.3%. Therefore, the effect of calculating the reservoir boundary distance of 7 m is the best when the source distance combination of 2 m and 4 m is used.

### 3.4. Ranging Effect at Different Dip Angles of Reservoir Boundary

When the drill collar drills into the reservoir, the reservoir dip angle will change accordingly. This change will have an impact on the distance measurement effect of the reservoir boundary. It is necessary to study the relative error of distance measurement under different dip angles.

The ranging method mentioned in Figure 9 and Equation (3) is only applicable to cases where the drill collar is parallel to the reservoir boundary. When the reservoir boundary has a dip angle, it needs to be re-analyzed by geometric methods.

**Figure 12:**
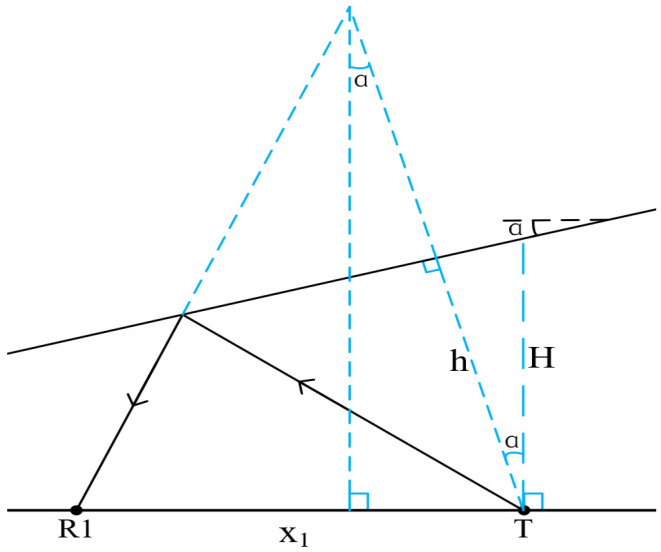
Inclined reservoir boundary schematic diagram.

Firstly, we consider the range method for a reservoir boundary with a dip angle. Assume that the included angle between the reservoir boundary and the drill collar is defined as positive when the reservoir boundary rotates counterclockwise, and negative otherwise. Similarly to Figure 9, the schematic diagram when the reservoir boundary dip angle is positive is shown in Figure 12.

In Figure 12, T is the sound source, R1 is a receiving point with a source distance of $x_{1}$ and a reflection wave arrival time of $t_{1}$. The formation velocity is *v*, and the distance from the sound source to the inclined interface is *h*. Under the condition of a homogeneous medium in the inclined interface, the reflection wave travel-time curve equation is as follows [32]:

(4) $$t_{1}=\frac{1}{v}\sqrt{x_{1}^{2}+4h^{2}-4hx_{1}\sin\alpha }$$

Derived from Equation (4),

(5) $$h=\frac{1}{2}\left(\sqrt{{\left(vt_{1}\right)}^{2}-x_{1}^{2}+{\left(x_{1}\sin\alpha \right)}^{2}}+x_{1}\sin\alpha \right)$$

**Figure 13:**
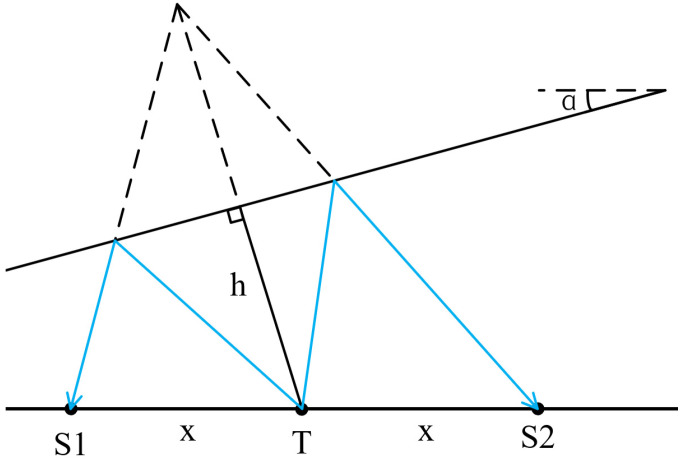
Formation velocity calculation schematic diagram.

In Equation (5), the formation velocity *v* can be derived and calculated from the schematic diagram shown in Figure 13.

Among them, *S1* and *S2* are receiving points on both sides of the sound source *T*, with the same source distance *x*. Their reflection wave travel times are $t_{s1}$ and $t_{s2},$ respectively, and the self-excited and self-received time at the sound source *T* is $t_{0}$. The calculation formula is as follows,

(6) $$t_{0}=\frac{2h}{v}$$

The time difference ∆t between the two receiving points can be approximated as [32],

(7) $$\Delta t=t_{2}-t_{1}=\frac{t_{0}x\sin\alpha }{h}$$

Substituting Equation (6) into Equation (7), the formation velocity *v* is obtained,

(8) $$v=\frac{2x\sin\alpha }{\Delta t}$$

Attaining *v* from Equation (8) and substituting it into Equation (5), we obtain the distance *h* from the sound source *T* to the inclined reservoir boundary. For the calculation and comparison of ranging accuracy, *h* is converted to *H* to facilitate comparative calculation. According to Figure 12, it can be easily seen that there is a triangular corresponding relationship, which is as follows,

(9) $$H=\frac{h}{\cos\alpha }$$

Using *H* obtained from Equation (9), the relative error of ranging when the reservoir boundary has a dip angle can be calculated.

**Figure 14:**
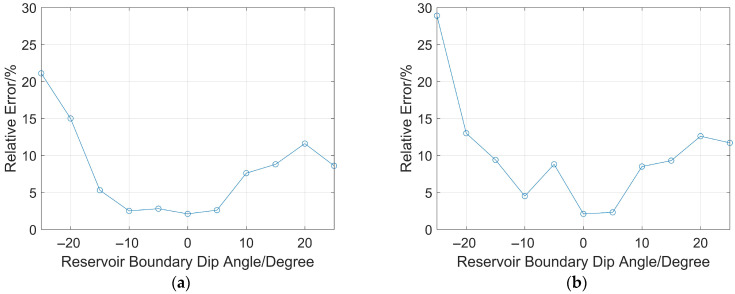
Relative error of ranging at different reservoir boundary dip angles: (**a**) source distance: 2 m; (**b**) source distance: 7 m.

Secondly, take the intersection point of the line from the impulse sound source *T* to the reservoir boundary (perpendicular to the drill collar) as the center, and rotate the reservoir boundary by 5°, 10°, 15°, 20°, 25°, −5°, −10°, −15°, −20°, and −25°. The counterclockwise rotation of the reservoir boundary is defined as positive, and the clockwise rotation is negative. Calculate the vertical distance between the sound source *T* and the reservoir boundary using Equations (5) and (8). The simulation benchmark remains H = 7 m, which is the distance from *T* to the parallel reservoir boundary. When the source distances are 2 m and 7 m, respectively (other conditions remain unchanged), the relative ranging errors at different rotation angles are shown in Figure 14a,b.

It can be observed from Figure 14a,b that when the source distance is 2 m, the relative error reaches a maximum of 21% when the reservoir boundary dip angle is −25°, while the relative ranging error does not exceed 15° when the dip angle is within ±20°; when the source distance is 7 m, the relative error peaks at 28% at a dip angle of −25°, and also does not exceed 15° when the dip angle is within ±20°. It is obvious that when both the reservoir boundary dip angle and the source distance are small, the reservoir boundary ranging method based on the impulse sound source has good ranging accuracy for inclined reservoir boundaries. However, when the reservoir boundary dip angle is large, it is necessary to integrate other ranging methods.

### 3.5. Ranging Effect Under Condition of Actual Formation Parameters

**Figure 15:**
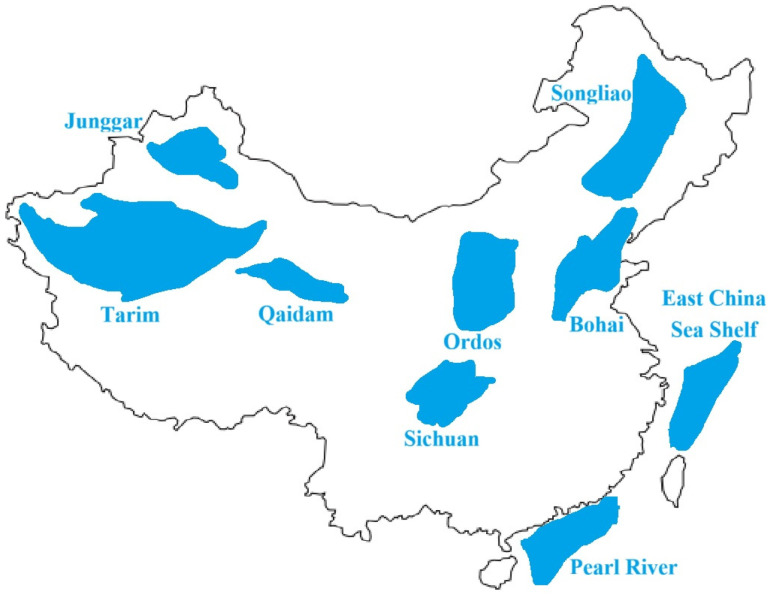
Schematic diagram of the distribution of China’s petroleum resources.

Shown in Figure 15 is a schematic diagram of the distribution of China’s petroleum resources. The spatial distribution of China’s petroleum resources exhibits distinct regional concentration characteristics, with such resources concentrated in the Tarim Basin, Junggar Basin, Qaidam Basin, Ordos Basin, Songliao Basin, Bohai Basin, Sichuan Basin, Pearl River Basin, and East China Sea Shelf Basin [33,34].

Tarim Basin and Junggar Basin are the two largest inland basins in China. The two basins are located, respectively, in the southern and northern parts of Xinjiang, China, with large oil and gas reserves. The two basins are important oil-producing areas in China; thus, their geological parameters are representative.

To verify the ranging effect of the impulse sound source in actual formations, the conventional logging data of the Jimusaer shale oil formation in Xinjiang [35] and the Shunbei Ordovician carbonate formation in the Tarim area [36] were referred to. Numerical simulation was conducted based on the formation data, with the specific formation data shown in Table 2, and the remaining model parameters using the data in Table 2.

**Figure 16:**
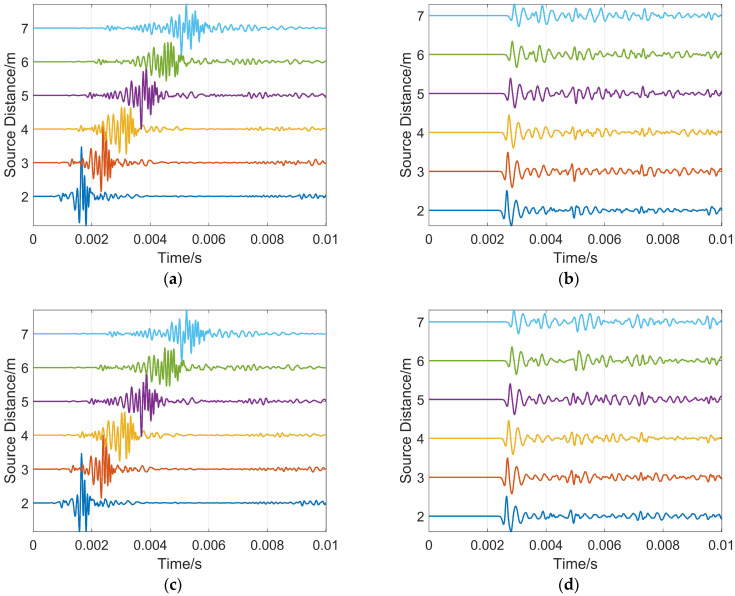
Full-wave train and reflected wave waveform diagrams: (**a**) Jimusaer formation full-wave train; (**b**) Jimusaer formation reflected wave; (**c**) Shunbei Ordovician formation full-wave train; (**d**) Shunbei Ordovician formation reflected wave.

The simulation model is shown in Figure 9. The reservoir boundary distance H = 7 m, the source distance combination is (2, 4), and the reservoir boundary dip angle is 0°. The full-wave train waveform diagrams were obtained from COMSOL simulation, as shown in Figure 16a,c, while the reflected wave waveforms extracted therefrom are shown in Figure 16b,d. The difference in sound pressure level between the full wave trains and the reflected waves is approximately 20–30 dB. As can be clearly observed from these drawings, the reflected waves are completely submerged by the full wave train signals, making them difficult to distinguish with the naked eye. Therefore, to achieve accurate ranging of reservoir boundaries, it is crucial to eliminate clutter interference and extract the reflected waves.

When the formation data is from the Jimusaer shale oil formation in Xinjiang, the peak arrival times of the reflected waves obtained with the source distance combination (2, 4) are 0.00266 s and 0.00274 s, respectively. Using Equation (3) for calculation, the distance from the reservoir boundary to the drill collar is 6.937 m, with a relative error of 0.90%. Similarly, when the formation data is from the Shunbei Ordovician carbonate formation in the Tarim area, the arrival times of the reflected waves are 0.00265 s and 0.00273 s, respectively, the distance from the reservoir boundary to the drill collar is 6.925 m, and the relative error is 1.07%.

From the simulation above, it can be concluded that the impulse sound source equipped with a rotational parabolic energy bunching reflector has a good capability of reservoir boundary ranging under actual formation parameters, and this ranging method has high accuracy. This method can meet the measurement requirements of both long distance and high accuracy, and is feasible for further in-depth application of impulse sound sources.

## 4. Conclusions

Through simulation research, this paper draws the following conclusions.

The energy bunching effect of a rotational parabolic energy bunching device is closely related to its own dimensions. Of these dimensions, the energy bunching effect is most sensitive to the depth of the device; thus, on the premise of ensuring drill collar strength, the depth should be prioritized. The opening radius of the device has a relatively minor impact on the sound pressure peak, so a smaller size can be selected to balance the requirement of drill collar strength. After equipping the parabolic reflector, the impulse sound source can gain azimuthal directivity, a characteristic that enables the impulse sound source to perform azimuthal reservoir boundary detection.

An impulse sound source equipped with this size of energy bunching reflector can achieve the measurement of the distance from the reservoir boundary to the drill collar. Through simulation, the capability of the impulse sound source for reservoir boundary distance measurement has been verified. Furthermore, verified under actual formation parameter conditions, this sound source demonstrates a capability for long-distance and high-precision boundary ranging, and has practical application prospects in LWD geological steering technology.

The research in this paper lays a foundation for subsequent in-depth research on impulse sound sources. This method can meet the measurement requirements of achieving both long distance and high accuracy, and can guide the wellbore trajectory to be close to oil-bearing reservoirs to achieve a higher horizontal well encounter rate.

Due to space constraints, this paper has not conducted relevant research on the relative ranging error under different sound source frequencies. Meanwhile, the theoretical verification of the impulse sound source-based ranging method has only been achieved via COMSOL simulation and the real-axis integration method. Future research will establish a physical experimental platform for further verification, and in light of the requirements for compact size and low power consumption of equipment under while-drilling operating conditions, develop adaptive compact, low-power-consumption impulse sound sources and energy bunching device structures.

## Figures and Tables

**Table 1:** Model parameters.

Material	$V\left(m/s\right)$	$\rho \;(kg/m^{3})$	Poisson’s Ratio	Young’s Modulus/Pa	r/m
Fluid Inside the Drill Collar	1500	1000			0.03572
Drill Collar		7850	0.28	$205\times 10^{9}$	0.0889
Fluid Outside the Drill Collar	1500	1000			0.10795
Formation		2500	0.2	$25\times 10^{9}$	
Reservoir		2600	0.25	$60\times 10^{9}$	

**Table 2:** Actual formation parameters.

Formation	$v_{p}\;(m/s)$	$v_{s}\;(m/s)$	$\rho \;(kg/m^{3})$
Jimusaer Shale Oil Formation in Xinjiang	6250	3310	2700
Shunbei Ordovician Carbonate Formation	6220	3450	2710
Tight Oil-bearing Reservoir	3810	2540	2500

**Figure A1:**
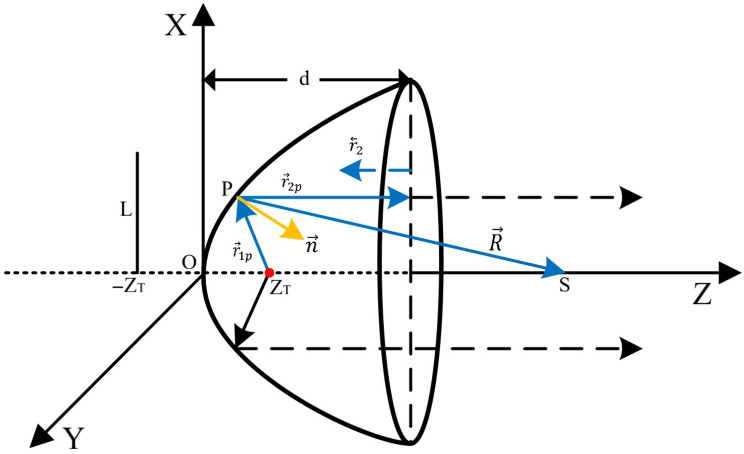
The geometric principle of the parabolic reflector.

## Data Availability

The original contributions presented in the study are included in the article; further inquiries can be directed to the corresponding author.

## Abbreviations

The following abbreviations are used in this manuscript:

PML	Perfectly Matched Layer
SNR	Signal-to-Noise Ratio
LWD	Logging While Drilling

## Appendix A

Here, based on the basic theory of acoustics, we analyze the time-domain analytical solution of the axial sound field of the paraboloid using Kirchhoff’s formula [28,37,38]. When the sound source is located at the apex of the paraboloid, the reflected wave field appears as a plane wave. Figure A1 is the geometric principle of the parabolic reflector bunching sound waves.

In Figure A1, the line *L* represents the directrix of the parabolic reflector. The point $P(X_{P},Y_{P},Z_{P})$ is any point on the curved surface, and ${\vec{r}}_{1p}$ is the distance between this point and the focus. ${\vec{r}}_{2p}$ represents the distance between any point on the curved surface and the opening of the surface, while ${\overset{\leftarrow }{r}}_{2}$ represents the distance between the opening of the surface and any point on ${\vec{r}}_{2p}$. The distance between the vertex of the reflector and the central point (0,0,d) of the surface opening is denoted as *d*, which is the depth of the reflector. $Z_{T}$ represents the distance between the vertex (0,0,0) of the curved surface and the focus $\left(0,0,Z_{T}\right)$, which is the focal length of the reflector. $\vec{n}$ represents the normal direction perpendicular to the inner side of the curved surface at point *P*. The distance between point *P* and any point $S(0,0,Z_{S})$ on the axis of the reflector is denoted as $\vec{R}$.

In the coordinate system as shown in Figure A1, Equation (A1) expresses the equation of the paraboloid:

(A1) $$X_{p}^{2}+Y_{p}^{2}=4Z_{T}Z_{p}(0<Z_{p}<d)$$

In the research on the impulse sound source energy bunching system, the observed physical quantity of the wave field is sound pressure. According to Kirchhoff’s formula for non-monochromatic waves, Equation (A2) is obtained:

(A2) $$P(\vec{r},t)=\frac{1}{4\pi }\iint_{s}\left\{〚p〛\frac{\partial }{\partial n}(\frac{1}{\vec{R}})-\frac{1}{\vec{R}c_{0}}\frac{\partial \vec{R}}{\partial n}〚\frac{\partial p}{\partial t}〛-\frac{1}{\vec{R}}〚\frac{\partial p}{\partial n}〛\right\}•dS$$

In Equation (A2), “⟦⟧” represents that the physical quantity inside the brackets experiences a time delay of $t-r/c_{0}$. According to the wave field propagation characteristics analyzed by Sommerfield [39], the changes in the wave field caused by sound waves emitted from the infinite surface $S^{\prime }$ can be ignored. So, the formula only needs the integral over the paraboloid. As long as the sound pressure distribution is given, we can calculate any sound pressure in *S* using Kirchhoff’s formula, without considering the secondary or multiple reflections inside the reflector.

When using a paraboloid as the reflecting structure of the impulse sound source, the relationship between the sound wave wavelength and the radius of curvature of the curved surface is what we need to focus on in this section. When their ratio is very small, we can use the theoretical basis of geometric acoustics to derive the boundary conditions of sound pressure on the curved surface [37]. First, we assume that the spherical wave radiated outward by the impulse sound source at the focus $Z_{T}$ is isotropic. Then we can use Equation (A3) to express the sound pressure at any point in space:

(A3) $$p(\vec{r},t)=\frac{A}{r}f(t-r/c_{0})$$

In Equation (A3), f(t) is the time-domain waveform function of the sound source, and *A* represents the sound pressure amplitude. We take a point at the focus $Z_{T}$ of the curved surface, and assume that the sound pressure amplitude at this point is $p_{o}$. So, for any point *P* on the curved surface, the corresponding sound pressure expression when the wavelength propagates to this point is as follows:

(A4) $$\frac{p(r_{1p},t)}{p_{0}}=\frac{Z_{T}}{r_{1p}}f(t-\frac{r_{1p}-Z_{T}}{c_{0}})$$

In Equation (A4), $r_{1p}=Z_{T}+Z_{S}$. We assume that when the wave field propagates to the surface of the parabolic reflector, total reflection of sound waves occurs on it. That is to say, we ideally consider the reflector structure to be rigid. We select the straight line $r_{2p}$, analyze the relationship between the sound pressure $p_{g}$ and the wave field propagation distance, and this can be expressed by Equation (A5):

(A5) $$\frac{p_{g}(r_{1p}+r_{2p}-r_{2},t)}{p_{0}}=\frac{Z_{T}}{Z_{T}+Z_{S}}f(t-\frac{d-r_{2}}{c_{0}})$$

where the subscript “*g*” represents the method of geometric acoustics. The spherical waves spreading from the sound source will have certain radiation directivity under the action of the reflector. Equation (A6) gives the definition of the corresponding directivity function:

(A6) $$D(Z_{S})=\left|\frac{p_{g}(Z_{S})}{p_{g}(0)}\right|=\frac{1}{1+\frac{Z_{S}}{Z_{T}}}$$

Combining Equations (A5) and (A6), we get the boundary conditions:

(A7) $${〚\frac{p_{g}}{p_{0}}〛}_{\;\;\;S}={\left.D(Z_{S})f(t-\frac{R+d-r_{2}}{c_{0}})\right|}_{r_{2}=r_{2p}}$$

In Equation (A7), the subscript “*S*” means that this value is taken on the paraboloid. Generally speaking, we cannot handle the time term when the sound source waveform f(t) is unknown. So, we fail to get the analytical solution by directly substituting Equation (A6) into Equation (A2). Based on the principle of linear systems, we can find the system output by convolving the input function with the system’s response transfer function. So, we can use the δ function as the system’s input. Then, the analytical solution of the sound field is obtained by convolving the sound source pulse waveform f(t) with h(t). Thus, Equation (A7) can be transformed into the following expression:

(A8) $${〚h_{g}〛}_{\;\;\;S}={\left.D(Z_{S})•\delta (t-\frac{R+d-r_{2}}{c_{0}})\right|}_{r_{2}=r_{2p}}$$

Substituting Equation (A8) into (A2), we get

(A9) $$h=\frac{1}{4\pi }\iint_{s}\left\{〚h_{g}〛\frac{\partial }{\partial n}(\frac{1}{\vec{R}})-\frac{1}{\vec{R}c_{0}}\frac{\partial \vec{R}}{\partial n}〚\frac{\partial h_{g}}{\partial t}〛-\frac{1}{\vec{R}}〚\frac{\partial h_{g}}{\partial n}〛\right\}•dS$$

Because the reflector has rotational symmetry, Equation (A10) is used to express the equation in the plane

(A10) $$C(X_{S},Z_{S})\to X_{S}^{2}-4Z_{T}Z_{S}=0$$

The unit vector of the inner normal of the parabolic reflector is

(A11) $${\vec{e}}_{n}=-\frac{\nabla_{S}C}{\left|\nabla_{S}C\right|}=-\frac{\sqrt{Z_{S}}{\vec{e}}_{X}-\sqrt{Z_{T}}{\vec{e}}_{Y}}{\sqrt{Z_{S}+Z_{T}}}$$

where the gradient operator $\nabla_{S}=\frac{\partial }{\partial X_{S}}{\vec{e}}_{X}+\frac{\partial }{\partial Z_{S}}{\vec{e}}_{Z}$. The distance between a point (0,Z) on the axis of the reflector and a point $\left(X_{S},Z_{S}\right)$ on the paraboloid $S_{0}$ is

(A12) $$R=\left|\vec{R}\right|=\sqrt{{\left(Z_{S}-Z\right)}^{2}+4Z_{T}Z_{S}}$$

At the same time, the directional derivative along the direction of the inner normal $\vec{n}$ of the paraboloid is

(A13) $$\frac{\partial \vec{R}}{\partial n}=\cos(\hat{\vec{n},\vec{R}})=-\frac{\sqrt{Z_{T}}(Z+Z_{S})}{R\sqrt{Z_{S}+Z_{T}}}$$

When taking a point along the axis of the reflector, each term on the right-hand side of Equation (A9) is, respectively

(A14) $$\left\{\begin{array}{c} {\left(〚h_{g}〛\frac{\partial }{\partial n}(\frac{1}{\vec{R}})\right)}_{\;\;\;\;S}=D(Z_{S})\frac{1}{R^{3}}\frac{\sqrt{Z_{T}(Z+Z_{S})}}{\sqrt{Z_{T}+Z_{S}}}\delta^{\prime (\tau )}\\ {\left(-\frac{1}{\vec{R}c_{0}}\frac{\partial \vec{R}}{\partial n}〚\frac{\partial h_{g}}{\partial t}〛\right)}_{\;\;\;\;S}=D(Z_{S})\frac{1}{c_{0}R^{3}}\frac{\sqrt{Z_{T}(Z+Z_{S})}}{\sqrt{Z_{T}+Z_{S}}}\delta^{\prime (\tau )}\\ dS=2\pi X_{S}\sqrt{1+{\left(\frac{dX_{S}}{dZ_{S}}\right)}^{2}dZ_{S}}=4\pi \sqrt{Z_{T}(Z_{T}+Z_{S})}•dZ_{S} \end{array}\right.$$

where $\tau =t-\frac{R+Z_{S}}{cc_{0}}$. Combining all the equations of Equation (A14), we get

(A15) $$h=\int_{0}^{d}D(Z_{S})\left[\frac{Z+Z_{S}}{R^{2}}\delta (\tau )+(\frac{Z+Z_{S}}{c_{0}R}+\frac{1}{c_{0}})\delta^{\prime }(\tau )\right]\frac{Z_{T}}{R}•dZ_{S}$$

By convolving f(t) and *h*, we get the sound pressure amplitude at any point on the axis of the paraboloid, and the corresponding analytical solution obtained from the operation is Equation (A16):

(A16) $$\frac{p(Z,t)}{p_{0}}=f(t)*h=f(t-\frac{Z}{c_{0}})+h_{e}f(t-\frac{R_{e}+d}{c_{0}})+\frac{c_{0}}{Z_{T}}\int_{\frac{S}{c_{0}}}^{\frac{R_{e}+d}{c_{0}}}h_{w}f(t-t^{\prime })•dt^{\prime }$$
